# The genomic and epigenomic landscape of iridocorneal endothelial syndrome

**DOI:** 10.1016/j.gendis.2024.101448

**Published:** 2024-11-06

**Authors:** Yaoming Liu, Gen Li, Jiaxuan Jiang, Sujie Fan, Lan Lu, Ting Wang, Guigang Li, Wenzong Zhou, Xuequn Liu, Yingjie Li, Hong Sun, Liang Liang, Yuhong Tang, Yang Chen, Jianjun Gu, Fei Li, Xiuli Fang, Tao Sun, Aiguo Lv, Yayi Wang, Peiyuan Wang, Tao Wen, Jiayu Deng, Yuhong Liu, Mingying Lai, Jingni Yu, Danyan Liu, Hua Wang, Meizhu Chen, Li Li, Xiaodan Huang, Jingming Shi, Xu Zhang, Kang Zhang, Lingyi Liang, Xiulan Zhang

**Affiliations:** a State Key Laboratory of Ophthalmology, Zhongshan Ophthalmic Center, Sun Yat-sen University, Guangdong Provincial Key Laboratory of Ophthalmology and Visual Science, Guangzhou, Guangdong 510060, China; b Guangzhou Women and Children’s Medical Center, Guangzhou Medical University, Guangzhou, Guangdong 510623, China; c Eye Hospital (The Third Hospital of Handan), Handan, Hebei 056000, China; d Department of Ophthalmology, Department of Ophthalmology & Optometry, Fujian Medical University, Fuzhou, Fujian 350004, China; e Eye Hospital of Shandong First Medical University, State Key Laboratory Cultivation Base, Shandong Provincial Key Laboratory of Ophthalmology, Shandong Eye Institute, Shandong First Medical University & Shandong Academy of Medical Science, Jinan, Shandong 250000, China; f Department of Ophthalmology, Tongji Hospital, Tongji Medical College, Huazhong University of Science and Technology, Wuhan, Hubei 430030, China; g Cangzhou Aier Eye Hospital, Cangzhou, Hebei 061000, China; h Nangchang Aier Eye Hospital, Nanchang, Jiangxi 330002, China; i The Third Affiliated Hospital of Nanchang University, Nanchang, Jiangxi 330008, China; j The First Affiliated Hospital of Nanjing Medical University, Nanjing, Jiangsu 210029, China; k Department of Ophthalmology, Yichang Central People’s Hospital, The First College of Clinical Medical Science, China Three Gorges University, Yichang, Hubei 443003, China; l Kunming Huashan Eye Hospital, Kunming, Yunnan 650032, China; m Kunming Aier Eye Hospital, Kunming, Yunnan 650041, China; n Affiliated Eye Hospital of Nanchang University, Jiangxi Research Institute of Ophthalmology and Visual Science, Nanchang, Jiangxi 330006, China; o Shenzhen Eye Hospital, Shenzhen, Guangdong 518000, China; p Department of Ophthalmology, Xi’an Fourth Hospital, Xi’an, Shaanxi 710004, China; q Department of Ophthalmology, Second Hospital of Hebei Medical University, Shijiazhuang, Hebei 050000, China; r Eye Center of Xiangya Hospital, Central South University, Hunan Key Laboratory of Ophthalmology, Changsha, Hunan 410008, China; s Department of Ophthalmology, The 900th Hospital of Joint Logistic Support Force, PLA (Clinical Medical College of Fujian Medical University, Dongfang Hospital Affiliated to Xiamen University), Fuzhou, Fujian 350025, China; t Department of Ophthalmology, The People’s Hospital Guangxi Zhuang Autonomous Region, Nanning, Guangxi 530021, China; u Eye Center, Second Affiliated Hospital, College of Medicine, Zhejiang University, Hangzhou, Zhejiang 310009, China; v The Second Xiangya Hospital of Central South University, Changsha, Hunan 410011, China; w Faculty of Medicine, Macau University of Science and Technology, Taipa, Macao 999078, China

**Keywords:** Copy number variance, DNA methylation, Etiology, Iridocorneal endothelial syndrome, Whole-genome sequencing

## Abstract

Iridocorneal endothelial (ICE) syndrome is a rare, irreversibly blinding eye disease with an unknown etiology. Understanding its genomic and epigenomic landscape could aid in developing etiology-based therapies. In this study, we recruited 99 ICE patients and performed whole-genome sequencing (WGS) on 51 and genome-wide DNA methylation profiling on 48 of them. We conducted mutational burden testing on genes and noncoding regulatory regions, comparing the ICE cohort with control groups (9197 East Asians from the gnomAD database and 350 normal Chinese from our in-house cohort). Copy number variation (CNV) analysis and differential methylation of regions were also explored. We identified RP1L1 (27/51, 53%) with a significantly higher coding-altering mutational burden in the ICE cohort (p < 8.3×10^−7^), with mutations predominantly at chr8:10467637 (hg19). Additionally, 41 regions with significant CNVs were identified, including two regions at chr19:15783859-15791329 (hg19) and chr3:75786061-75790887 (hg19), showing copy number loss in 39 and 19 patients, respectively. We also identified 2,717 differentially methylated regions (DMRs), with hypomethylation prevalent in ICE syndrome (91.9% of DMRs). Among these, 45 recurrent hypomethylated regions (HMRs) in more than 10% of ICE patients showed differential methylation compared to normal controls. This study presents the first comprehensive genomic and epigenomic characterization of ICE syndrome, offering insights into its underlying etiology.

## Introduction

The iridocorneal endothelial (ICE) syndrome is a rare but irreversibly blinding ocular disorder characterized by the proliferation of abnormal corneal endothelial cells that migrate toward the iridocorneal angle and iris surface, leading to corneal decompensation, glaucoma secondary to peripheral anterior synechiae, and ultimately, vision loss.^1^ It comprises a spectrum of clinical entities: progressive essential iris atrophy, Cogan-Reese syndrome, and Chandler syndrome. More than half of ICE syndrome patients show progressive development with secondary glaucoma or corneal endothelial dysfunction,^2^, ^3^, ^4^ while symptomatic anti-glaucoma surgery and corneal transplant surgery have unsatisfying prognosis and high relapse rates.^5^, ^6^, ^7^

Herpes simplex viral DNA has been reportedly recovered from the cornea of ICE patients,^8^^,^^9^ whereas, HSV-1 DNA could be obtained in cornea and aqueous humor among patients without a history of ocular herpes, which indicates that latent ocular herpes virus is very common.^10^^,^^11^ Alternatively, a hypothesis was proposed that the ICE syndrome is the result of an altered proliferation of neural crest cells from which corneal endothelial cells derive,^12^ and the possibility of embryonic ectopia of ocular surface epithelium as the origin of abnormal ICE cells was also contemplated.^13^^,^^14^ Additionally, it was postulated that ICE cells could arise from a metaplastic stimulus, or ocular pathology such as a viral infection, which is required for the embryonic ectopia to proliferate.^15^ However, none of these hypotheses can completely explain the exact pathogenesis, and neither could they be verified in animal models or humans. The debate on ICE syndrome’s etiology is still ongoing after more than a century, and therefore, there is still a lack of targeted effective drugs for ICE syndrome due to its largely unknown etiology.

The discrepancy in the most common ICE syndrome variant among different studies suggests ethnic differences and susceptibilities.^2^^,^^16^^,^^17^ Recent studies suggest that epigenetic modifications play a role in the manifestation of another corneal endothelial disease, Fuchs endothelial corneal dystrophy and differentially methylated probes have been identified to affect proteins involved in cytoskeletal organization, ion transport, and cellular metabolism in this disease.^18^^,^^19^ However, no study has ever characterized genomic predisposition and epigenomic alterations in ICE syndrome. Given the success of unbiased next-generation sequencing in identifying pathogenic mutations and epigenetic changes in many disorders,^20^ we recruited a cohort of affected individuals from multiple medical institutes in China and performed exhaustive whole-genome sequencing on 51 patients and genome-wide DNA methylation profiling on 48 patients. In this study, we provide the first comprehensive genomic and epigenomic landscape of ICE syndrome, which will shed light on the development of its etiology-based therapies.

## Patients and methods

### ICE syndrome cohort and control cohort

The study was approved by the Institutional Review Board at Zhongshan Ophthalmic Center (2017KYPJ071) and other participating centers. Informed consent was obtained from affected individuals participating in the study. We recruited a cohort of 99 affected individuals with a clinical diagnosis of ICE syndrome from 20 medical institutes in China (Table S1). The diagnosis of ICE syndrome was based on typical ocular findings on the cornea (slit lamp photograph, corneal endothelium specular microscopy, and/or *in vivo* confocal microscopy) and iris (slit lamp photograph).^1^ All three clinical variants of ICE including Chandler syndrome, progressive iris atrophy, and Cogan-Reese syndrome were included in this study (Fig. S1). All diagnoses made in different centers were confirmed by two doctors at Zhongshan Ophthalmic Center. According to our study protocol, incidental findings that were unrelated to clinical features at presentation were not reported.

The resultant variant call data (in variant call file/VCF format) of the control group were collected from the gnomAD database^21^ (https://gnomad.broadinstitute.org/, including 9197 East Asians with whole-exome sequencing data and 780 East Asians with whole-genome sequencing data) and our in-house cohort (including 350 normal Chinese with whole-genome sequencing data). The methylation data of the control group were collected from our in-house cohort (including 45 normal Chinese with genome-wide DNA methylation data).

### Whole-genome sequencing

DNA from whole blood samples was obtained for all individuals using E.Z.N.A.® Blood DNA Midi Kit (Omega Bio-tek, Inc., Norcross, USA) according to the manual, and was then sent to Novogene Bioinformatics Technology Co., Ltd (Novogene, Beijing, China) for whole-genome sequencing. Illumina Paired Library preparation was done following the manufacturer’s instructions (Illumina, San Diego, CA, USA), which was used for whole-genome amplification, and sequencing was carried out on an Illumina HiSeq X Ten platform, with 2 × 150 bp paired-end reads. Reads were pre-processed with Trim Galore to remove sequencing adapters and to filter the sequences with a minimum Phred quality score of 20.

### Variant calling and annotation

We performed joint variant calling for variants across all samples in this cohort using GATK v4.1.4 (https://gatk.broadinstitute.org/hc/en-us). Specifically, we used the HaplotypeCaller pipeline according to GATK best practices. Variant quality score recalibration (VQSR) was performed, and only “PASS” variants were investigated. The resultant VCF was annotated with Variant Effect Predictor v102.0,^22^ Loftee,^21^ dbNSFP,^23^ and Annovar.^24^ Variants with an allele frequency of ≤0.01 in gnomAD were considered rare. Cohort quality control including ancestry analysis, crypic relatedness, and sex checks was performed using peddy.^25^ Specifically, PCA was performed on 1000 Genomes project samples for the overlap of variants measured in the ICE cohort with ∼25,000 variants from samples in the 1000 Genomes project. ICE cohort samples were then projected onto these PCs, and ancestry in the ICE cohort was predicted from the PC coordinates using a support vector machine trained on known ancestry labels from 1000 Genomes samples. Relatedness parameters were calculated (coefficient of relatedness, ibs0, ibs1, ibs2) using these variants and were compared to known relationships from the cohort pedigrees; cases that did not agree were manually validated and corrected. In all cases, sex checks (presence of heterozygous variants on the X chromosome) performed by peddy aligned with available cohort information.

### Gene- and regulatory segment-based burden analyses

Gene- and regulatory segment-based burden testing was performed using TRAPD,^26^ which employs a one-sided Fisher’s exact test of the 2 × 2 table of genotype counts (genotype present or genotype absent) between case subjects (51 unrelated case subjects from the ICE cohort) and control subjects. GnomAD v2.1 is an aggregation database of genome sequencing from 123,136 individuals who are not known to have a severe Mendelian condition, which includes 9197 exomes and 780 genomes of East Asians.^27^ Counts under the dominant model were generated for ICE by counting the number of individuals who carry at least one qualifying variant in each gene and for gnomAD by summing the allele counts for qualifying variants in each gene or regulatory segment. To explore mutation burdens within noncoding regulatory segments (promoter, enhancer, 5′ UTR, 3′ UTR, lncRNA, lncRNA promoter, miRNA, and small RNA; bed files were obtained from the study of the Pan-Cancer Analysis of Whole Genomes (PCAWG) Consortium)^28^ in ICE syndrome. We defined deleterious noncoding mutations when i) max gnomAD allele frequency ≤0.001; ii) predicted as deleterious by all the 3 algorithms: GERP++,^29^ GWAVA,^30^ and CADD.^31^ Several steps were taken to match variant call set quality since variants were not jointly called for the case and control subjects. First, read depth was computed in each cohort separately and only bases with at least 90% of samples covered in both ICE and gnomAD were retained for analyses. Second, sites present in low-complexity regions were removed. Third, rare benign variant burden testing was performed for different VQSR combinations until the -log_10_(*P* value) from Fisher’s exact test followed the expected distribution.

### Copy number variance analysis

Copy number variant (CNV) calling was first performed using the software package Control-Freec (v7.0)^32^ and CNVnator (v0.4.1).^33^ Regions with significant CNVs reported by both Control-Freec and CNVnator were retained and merged. Calls were merged if they overlapped reciprocally by 50%, and then for each pair of calls, we averaged the coordinates of the start and coordinates of the end. Merged regions with significant CNVs were then analyzed and compared between the ICE cohort and our in-house normal Chinese cohort using the software package coverageMaster (v1.1).^34^

### Library construction and genome-wide DNA methylation profiling

DNA from whole blood samples was obtained for all individuals using E.Z.N.A.® Blood DNA Midi Kit (Omega Bio-tek, Inc., Norcross, USA) according to the manual. The library was prepared by using the TruSeq Methyl Capture EPIC Library Prep Kit (TMC-EPIC kit, FC-151-1003, Illumina) according to the manufacturer’s instructions except that the fragmentation step was skipped over. The screening regions covered in the TMC-EPIC Kit were indicated in supplementary data, with a coverage of over 3.3 million CpG sites, largely exceeding the commonly utilized 450K or 850K assays, which surveyed 45,000 and 85,000 CpG sites, respectively. The concentration of prepared libraries was determined by the Qubit 2.0 fluorometer (Invitrogen, Life Technologies, USA), and the libraries' quality was assessed by capillary electrophoresis (Qsep100, BiOptic, Taiwan, China). Qualified libraries were sequenced on the Illumina HiSeq X10 platform (Illumina, USA).

### DNA methylation data processing and analysis pipeline

Alignment, trimming, and methylation calling were performed through the default workflow using Bismark^35^ with Bowtie2^36^ as an alignment tool in the nf-core/methylseq pipeline (v1.6.1, https://github.com/nf-core/methylseq).^37^ Ten bases were trimmed off the 5′ end of every read per the Bismark User Guide recommendations for the library kit used. Alignment to GRCh37, de-duplication, and base-level methylation calling were performed with Bismark 0.23.0 using the default parameters as recommended by the Bismark User Guide for the library kit. The “--paired-end” and “--no_overlap” parameters were set. Hypomethylated regions (HMRs) were identified using MethylSeekR 1.30.0 using the default parameters.^38^ Only bases with a minimum coverage of 5 reads (the default MethylSeekR cutoff) were included for subsequent analysis. Regional annotation of HMRs was carried out by Annovar,^24^ including regions of promoters, enhancers, dyadic regions, and CpG islands/shores/shelves.^39^ DMRs (differentially methylated regions) were analyzed via the student’s *t*-test. Functional enrichment analysis of genes on DMRs was realized through clusterProfiler.^40^ Recurrent HMRs were defined by running a 100 bp sliding window across the genome and identifying contiguous regions where MethylSeekR was called an HMR in ≥5% of samples.^39^ Only focal HMRs (≤10 kb) were utilized in this analysis. HMRs were assigned to the first group that overlapped in the following order: promoter, enhancer, and dyadic regions.

## Results

### Overall yield and spectrum of variants in the ICE cohort

To comprehensively identify risk mutations and genes, we investigated rare coding-altering, loss of function, and missense mutations in genes, called CNVs using whole-genome sequencing coverage estimates, and performed gene burden analyses to nominate new genes. Whole-genome sequencing was performed on 51 cases (39 females and 12 males; Table S2) with diagnoses of Chandler syndrome (*n* = 23; Fig. S1A), progressive iris atrophy (*n* = 20; Fig. S1B), and Cogan-Reese syndrome (*n* = 8; Fig. S1C) with an average depth of 28.87 ± 5.52. The average genome coverage was 99.65% ± 0.34%. Cohort quality control analysis showed that the ancestry of samples in the ICE cohort was classified as East Asian as expected (Fig. S2A). Relatedness parameters confirmed the unrelatedness among individuals in our cohort.

Single nucleic variants and indels ratios were significantly higher in all noncoding regulatory segments (from 15.15 ± 0.98 to 20.46 ± 4.44 mutations per Mb) compared with coding regions (14.70 ± 0.66 mutations per Mb; *t*-test, all false discovery rates <0.05) (Fig. S2B, C). Within noncoding regulatory segments, mutation ratios were significantly higher in small RNA and miRNA regions (20.46 ± 4.44 mutations per Mb; 19.83 ± 10.66 mutations per Mb) compared with other noncoding areas (from 15.15 ± 0.98 to 16.47 ± 0.89 mutations per Mb; *t*-test, all false discovery rates <0.05) (Fig. S2B), and mutational burden variations were much larger in small RNA and miRNA regions possibly due to their smaller lengths. Most aberrant genomic variants were unique, with 80% of mutations observed in not more than one unrelated case.

### RP1L1 has a higher coding-altering mutational burden in the ICE syndrome cohort

**Figure 1:**
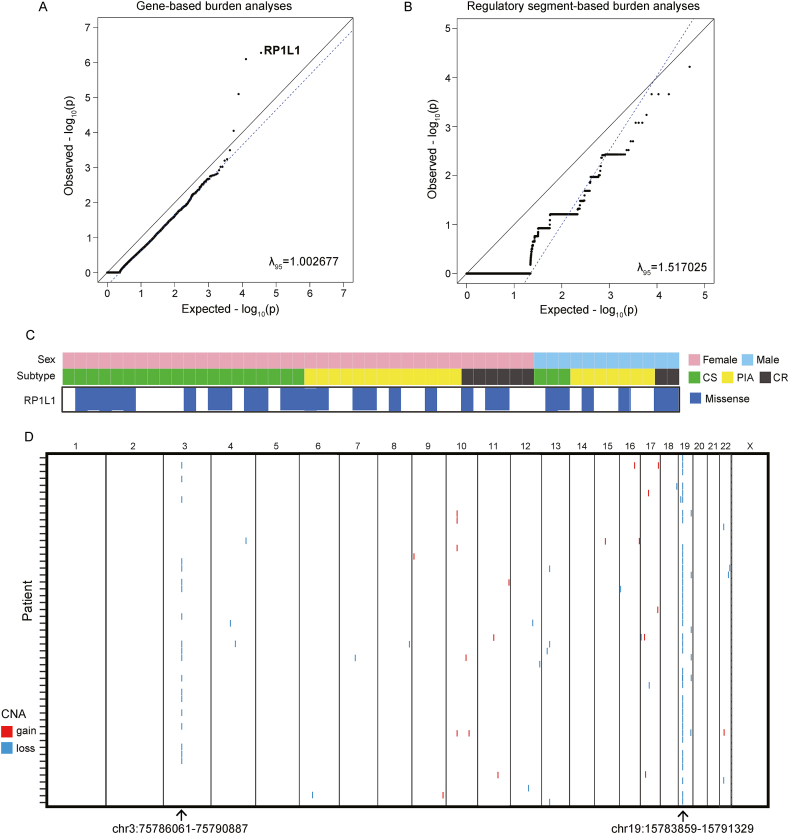
Mutational landscape of iridocorneal endothelial (ICE) syndrome. **(A)** Gene-based burden test of rare coding-altering mutations (max gnomAD AF ≤ 0.01). **(B)** Mutation burden analysis of rare pathogenic noncoding mutations (max gnomAD AF ≤ 0.001; predicted deleterious by all the 3 algorithms: GERP++, GWAVA, and CADD). **(C)** Gene RP1L1 was identified with a significantly coding-altering mutational burden. **(D)** Copy number variations identified throughout chromosomes in ICE patients. CS, Chandler syndrome; PIA, progressive iris atrophy; CR, Cogan-Reese syndrome.

We sought to identify novel genes associated with ICE syndrome by performing gene burden tests between unrelated individuals in our cohort and gnomAD control subjects (a cohort presumably depleted of rare pediatric diseases), and the significant results were then filtered by comparison between ICE cohorts and our in-house cohorts of 350 normal Chinese. We first carefully adjusted the variant quality thresholds between the case and control subjects such that no genes were more enriched for synonymous mutations (max gnomAD allele frequency ≤0.01) in the ICE cohort than expected. Specifically, the inclusion of the top 95% quality-by-depth of VQSR variants from the ICE cohort and all VQSR variants from the gnomAD cohort resulted in the best (Fig. S2D; λ_95_ = 1.03). When performing gene-based burden testing of rare coding-altering mutations (max gnomAD allele frequency ≤0.01) between the ICE syndrome cohort and normal control on the dominant model, gene RP1L1 (retinitis pigmentosa 1-like 1) was found to have a higher mutation burden in the ICE cohort (27/51) than in gnomAD (735/9197, *P* = 1.67 × 10^−13^) (Fig. 1A and Table S3) and our in-house normal Chinese cohort (62/350, *P* = 2.06 × 10^−7^) (Table S3).

To explore mutation burdens within noncoding regulatory segments (including areas of promoter, enhancer, 5′ UTR, 3′ UTR, lncRNA, lncRNA promoter, miRNA, and small RNA) in ICE syndrome, we first measured inflation with rare benign noncoding mutations (max gnomAD allele frequency ≤0.001; not predicted deleterious by any of the 3 algorithms: GERP++, GWAVA, and CADD), and inclusion of the top 95% quality-by-depth of noncoding variants from the ICE cohort and all of noncoding variants from the gnomAD cohort also resulted in the best (Fig. S2E; λ_95_ = 1.02). Then, we performed burden testing of rare pathogenic mutations (max gnomAD allele frequency ≤0.001; predicted deleterious by all the 3 algorithms: GERP++, GWAVA, and CADD) within the noncoding regulatory area, but none of the noncoding regulatory segments reached significant association (Fig. 1B).

### Copy number alterations in ICE syndrome

We performed whole-genome sequencing-based CNV calling and identified 634 significant CNVs (489 CNVs of loss, and 145 CNVs of gain) called by both Control-Freec and CNVnator across all ICE patients. These merged regions with significant CNVs were then analyzed and compared between the ICE cohort and our in-house normal Chinese cohort via coverageMaster, and we identified 41 regions exhibiting significant CNVs within the ICE syndrome cohort (Fig. 1C and Table 1; Table S4). Specifically, the segment at chr19: 15783859-15791329 (hg19) displayed copy number loss in 39 out of 51 ICE patients, and the region at chr3:75786061-75790887 (hg19) showcased copy number loss in 19 out of 51 ICE patients.

**Table 1:** Top 6 significant copy number variants identified in the ICE syndrome cohort.

Chr	Start	End	Cnvtype	Patient frequency	Cnv_length	CytoBand	Overlaped_genes
19	15783859	15791329	DEL	39	7469	p13.12	CYP4F12
3	75786061	75790887	DEL	19	4825	p12.3	ZNF717; MIR4273
19	55276166	55295820	DEL	5	19653	q13.42	LOC101928804; KIR2DL1
10	46995904	47000935	DUP	4	5030	q11.22	GPRIN2
13	32533646	32533721	DEL	3	74	q13.1	EEF1DP3
22	17264339	17264882	DEL	2	542	q11.1	XKR3

### Epigenomic alterations in ICE syndrome

To comprehensively identify epigenomic alterations, we conducted epigenomic screening on 48 cases of ICE syndrome, which covers over 3.3 million CpG sites, far exceeding the coverage of commonly utilized 450K and 850K assays which only surveyed 45,000 and 85,000 CpG sites, respectively. The total number of HMRs found ranged from 14,939 to 37,156 per sample (Fig. S3A). Averagely 63.2% of HMRs were on promoters and others located in regulatory regions such as enhancer sites (33.3%) and dyadic (promoter/enhancer) regions (2.0%) (Fig. S3A). DNA methylation has been best characterized at the CpG islands present in promoter regions of genes.^41^, ^42^, ^43^ However, on average only 23.6% of HMRs located on CpG islands/shores/shelves (Fig. S3A), and 77.2% (32,030/41,501) of the recurrent HMRs (present in ≥5% of samples) were found outside of CpG islands, shores, or shelves (Table S5). We hypothesized that recurrent intergenic HMRs would be associated with regulatory loci. We found that 40.6% of recurrent HMR sites overlapped promoter sites, and 53.6% of recurrent HMR sites only overlapped enhancer regions rather than promoters.

**Figure 2:**
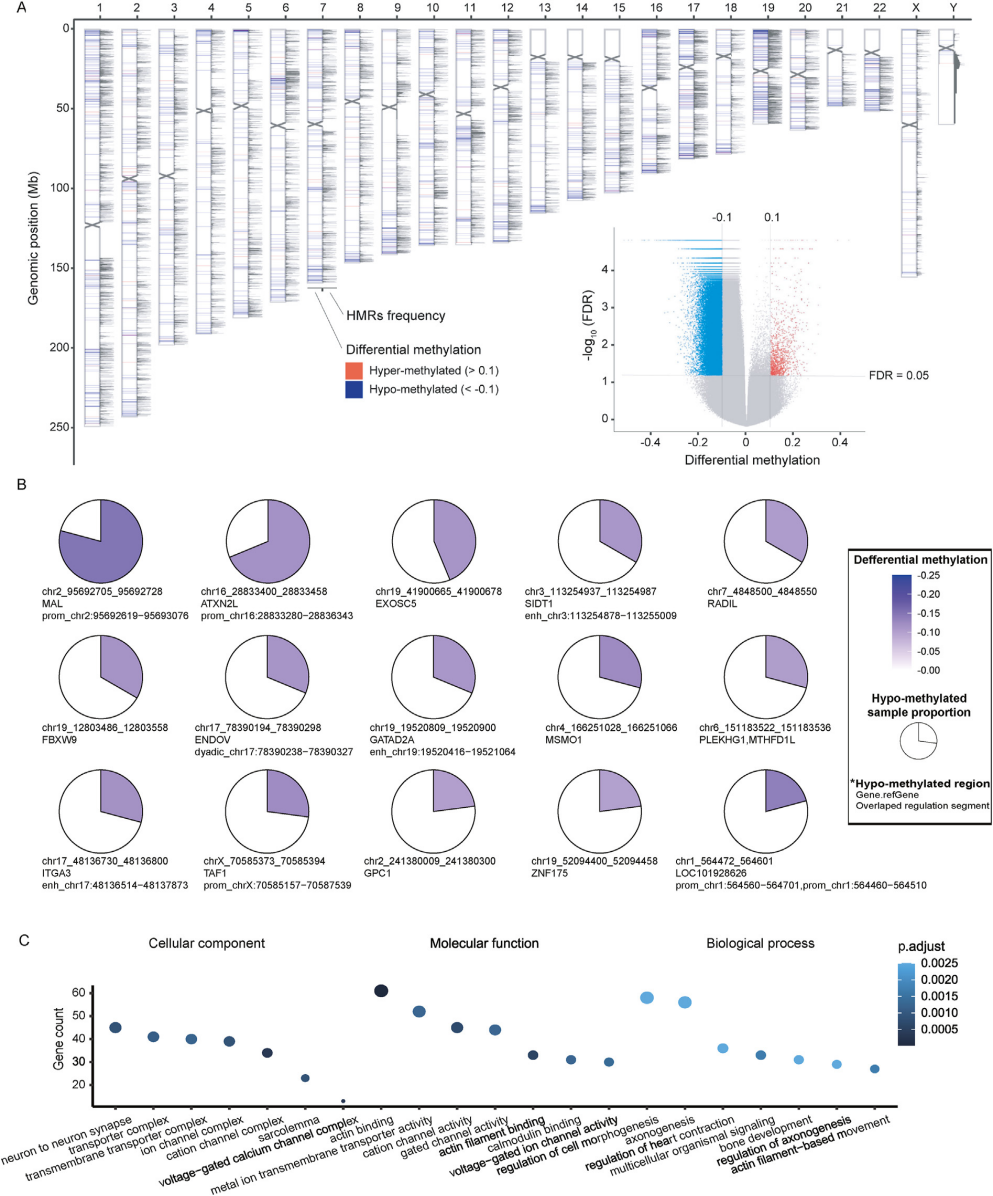
Epigenomic alterations of iridocorneal endothelial (ICE) syndrome. **(A)** Differentially methylated region (DMR) and hypomethylated region (HMR) frequency in ICE syndrome. The ideogram shows, for each chromosome, from left to right: DMR comparing ICE syndrome (*n* = 48) to normal Chinese (*n* = 45), and HMR frequency in 100-bp windows in the ICE samples. The volcano plots show differentially methylated loci comparing ICE syndrome to normal Chinese. **(B)** The top 15 recurrent HMRs among >10% of ICE patients also showed differential methylation (<−0.1) versus normal Chinese controls. **(C)** Gene Ontology analysis of differentially methylated genes. Seven significantly enriched terms of biological processes, cell components, and molecular functions are shown, respectively. The adjusted *P*-value was set to 0.05.

We then identified DMRs by comparing genome-wide DNA methylation data on ICE syndrome versus our in-house normal Chinese. ICE syndrome was predominantly less methylated than normal Chinese (in 91.9% of 2717 DMRs; Fig. 2A and Table S6). We also found 45 recurrent HMR regions (identified in >10% ICE samples tested) which were differentially methylated (differential methylation < −0.1) between ICE syndrome and normal Chinese (Table S7). Differentially methylated genes (DMGs) were defined as genes whose promoter, intron, or exon regions overlap with DMRs. Apart from the overlap, we found approximately 1432 DMGs in total. To probe the gene functions of DMGs exposed to ICE syndrome, GO pathway analyses were carried out to characterize the DMGs. The GO analysis showed that DMGs were significantly enriched in multiple biological processes, such as actin binding, metal ion transmembrane transporter activity, cation channel activity, regulation of cell morphogenesis, and axonogenesis (Fig. 2C). These results suggested that these DMGs might affect cell morphogenesis and metal ion transportation, subsequently contributing to corneal endothelial dysregulation and the pathogenesis of ICE syndrome.

## Discussion

The etiology of ICE syndrome urgently needs to be revealed. Whether there are genetic predispositions and epigenomic alterations in ICE syndrome remains unknown. Our study recruited an ICE syndrome cohort, performed whole-genome sequencing and genome-wide DNA methylation profiling, and characterized the barely known genomic and epigenomic landscape of ICE syndrome for the first time.

We explored genetic predispositions in ICE syndrome via whole-genome sequencing in 51 ICE patients compared with East Asians and normal Chinese. Considering rare coding-altering mutation burden with dominant inheritance, we identified RP1L1 as significantly associated with ICE syndrome. Referring to noncoding regulatory, none was identified as significantly associated with ICE syndrome. RP1L1 encodes for a protein that is part of the photoreceptor axoneme, and RP1L1 variants are associated with a spectrum of inherited retinal diseases including retinitis pigmentosa and occult macular dystrophy.^44^ The role of RP1L1 in the pathogenesis of ICE syndrome needs to be further investigated.

Then we investigated copy number variance in ICE syndrome, and the regions at chr19: 15783859-15791329 (hg19), chr3:75786061-75790887 (hg19), and chr19: 55276166-55295820 (hg19) showcased copy number loss in 39 cases, 19 cases, and 5 cases out of 51 ICE patients, respectively. Gene *CYP4F12* (cytochrome P450 family 4 subfamily F member 12) located on chr19: 15783859-15791329 (hg19) was reported to be associated with Bietti corneoretinal crystalline dystrophy, which is characterized by crystalline deposits in the retina and cornea.^45^^,^^46^ This indicated that copy number loss of gene *CYP4F12* may destroy oxidation by cytochrome P450 and thereby induce dysfunction of corneal endothelial cells in ICE syndrome. Gene *ZNF717* (zinc finger protein 717) located on chr3:75786061-75790887 (hg19) encodes a transcriptional regulator, involved in cell proliferation and regulation of viral replication and transcription, which was reported to be associated with HBV-related hepatocellular carcinoma.^47^ Gene *KIR2DL1* (killer cell immunoglobulin-like receptor, two Ig domains and long cytoplasmic tail 1) located on chr19: 55276166-55295820 (hg19) was reported to be associated with Behcet syndrome and viral infection.^48^, ^49^, ^50^ This indicated that copy number loss of gene *ZNF717* and *KIR2DL1* may lead to viral infection-induced pathological conversion of corneal endothelial cells in ICE syndrome.

We have identified an epigenetic landscape of ICE syndrome that is characterized by hypomethylation both within and outside CpG islands, shores, and shelves. Similar to cancers which are characterized by genome-wide hypomethylation and site-specific hypermethylation,^51^ genome-wide hypomethylation was also found in ICE syndrome patients in our study (in 91.9% of 2717 DMRs). This cancer-like genome-wide hypomethylation might be related to gain-of-function changes which affect cell cycle arrest of corneal endothelial cells in ICE syndrome.^52^ Differentially methylated genes were significantly enriched in multiple biological processes, such as actin binding, metal ion transmembrane transporter activity, and regulation of cell morphogenesis, which may contribute to corneal endothelial dysregulation and dysmorphism in the pathogenesis of ICE syndrome. We also found 45 recurrent HMR regions (identified in >10% ICE samples tested) that were differentially methylated (differential methylation < −0.1) between ICE syndrome and normal Chinese. Chr2_95692705_95692728 (hg19), the most frequent HMR identified in ICE patients, lies on the promotor of gene *MAL*. MAL functions in apical transport and membrane signaling in polarized epithelial cells, whose methylation and expression changes have been found involved in various epithelial oncogenesis.^53^^,^^54^ This indicated that promoter hypomethylation and elevated expression of MAL may contribute to epithelioid hyperplasia of corneal endothelial cells in ICE syndrome.

To summarize, the genes showing increased mutation burden, copy number variations, or hypomethylation that we have identified in ICE syndrome patients are primarily associated with pathogenic genes related to ocular diseases (*e.g.*, *RP1L1*, *CYP4F12*, and *KIR2DL1*), viral immunity (*e.g.*, *ZNF717* and *KIR2DL1*), and epithelioid hyperplasia (*e.g.*, *MAL*). This suggests that viral infections may trigger the pathological transformation of corneal endothelial cells based on these genetic and epigenetic susceptibilities, leading to ICE syndrome development. Our study represents the first comprehensive genomic and epigenomic analysis of ICE syndrome using unbiased next-generation sequencing with the largest sample size to date (99 cases). Although this sample size is substantial for a rare disease like ICE syndrome, larger cohorts could unveil additional pathogenic genomic and epigenomic variations. Furthermore, further validations and functional experiments are crucial to unravel the underlying mechanisms of ICE syndrome, which could pave the way for etiology-based therapeutic strategies.

## Web resources

GnomAD Browser, https://gnomad.broadinstitute.org/; GATK, https://gatk.broadinstitute.org/; Peddy, https://github.com/brentp/peddy; VEP, https://asia.ensembl.org/info/docs/tools/vep/index.html; ANNOVAR, https://doc-openbio.readthedocs.io/projects/annovar/en/latest/; TRAPD, https://github.com/mhguo1/TRAPD; Control-FREEC, http://boevalab.inf.ethz.ch/FREEC/; CNVnator, https://github.com/abyzovlab/CNVnator; coverageMaster, https://github.com/fredsanto/coverageMaster; AnnoteSV, https://www.lbgi.fr/AnnotSV/; nf-core/methylseq, https://github.com/nf-core/methylseq; MethylSeekR, https://bioconductor.org/packages/release/bioc/html/MethylSeekR.html.

## CRediT authorship contribution statement

**Yaoming Liu:** Conceptualization, Data curation, Formal analysis, Investigation, Methodology, Project administration, Software, Validation, Visualization, Writing – original draft, Writing – review & editing. **Gen Li:** Data curation, Formal analysis, Investigation, Methodology, Software, Validation, Visualization, Writing – original draft, Writing – review & editing. **Jiaxuan Jiang:** Data curation, Formal analysis, Investigation, Project administration, Validation. **Sujie Fan:** Data curation, Investigation, Resources, Writing – review & editing. **Lan Lu:** Data curation, Investigation, Resources, Writing – review & editing. **Ting Wang:** Data curation, Investigation, Resources, Writing – review & editing. **Guigang Li:** Data curation, Investigation, Resources, Writing – review & editing. **Wenzong Zhou:** Data curation, Investigation, Resources, Writing – review & editing. **Xuequn Liu:** Data curation, Investigation, Resources, Writing – review & editing. **Yingjie Li:** Data curation, Investigation, Resources, Writing – review & editing. **Hong Sun:** Data curation, Investigation, Resources, Writing – review & editing. **Liang Liang:** Data curation, Investigation, Resources, Writing – review & editing. **Yuhong Tang:** Data curation, Investigation, Resources, Writing – review & editing. **Yang Chen:** Data curation, Investigation, Methodology, Project administration, Validation, Writing – review & editing. **Jianjun Gu:** Data curation, Investigation, Resources, Writing – review & editing. **Fei Li:** Conceptualization, Data curation, Investigation, Writing – review & editing. **Xiuli Fang:** Data curation, Investigation, Methodology, Validation, Writing – review & editing. **Tao Sun:** Data curation, Investigation, Resources, Writing – review & editing. **Aiguo Lv:** Data curation, Investigation, Resources, Writing – review & editing. **Yayi Wang:** Data curation, Investigation, Writing – review & editing. **Peiyuan Wang:** Data curation, Investigation, Validation, Writing – review & editing. **Tao Wen:** Data curation, Investigation, Validation, Writing – review & editing. **Jiayu Deng:** Data curation, Investigation, Methodology, Writing – review & editing. **Yuhong Liu:** Data curation, Investigation, Methodology, Writing – review & editing. **Mingying Lai:** Data curation, Investigation, Resources, Writing – review & editing. **Jingni Yu:** Data curation, Investigation, Resources, Writing – review & editing. **Danyan Liu:** Data curation, Investigation, Resources, Writing – review & editing. **Hua Wang:** Data curation, Investigation, Resources, Writing – review & editing. **Meizhu Chen:** Data curation, Investigation, Resources, Writing – review & editing. **Li Li:** Data curation, Investigation, Resources, Writing – review & editing. **Xiaodan Huang:** Data curation, Investigation, Resources, Writing – review & editing. **Jingming Shi:** Conceptualization, Data curation, Investigation, Resources, Writing – review & editing. **Xu Zhang:** Data curation, Investigation, Project administration, Resources, Supervision, Writing – review & editing. **Kang Zhang:** Conceptualization, Data curation, Formal analysis, Funding acquisition, Investigation, Methodology, Project administration, Resources, Supervision, Validation, Writing – original draft, Writing – review & editing. **Lingyi Liang:** Data curation, Investigation, Project administration, Resources, Supervision, Writing – review & editing. **Xiulan Zhang:** Conceptualization, Data curation, Formal analysis, Funding acquisition, Investigation, Methodology, Project administration, Resources, Supervision, Validation, Writing – original draft, Writing – review & editing.

## Funding

This research was supported by the 10.13039/501100012166National Key Research and Development Program of China (No. 2022YFC2502800) and the 10.13039/501100001809National Natural Science Foundation of China (No. 82070955, 32000537).

## Conflict of interests

The authors declared no competing interests.

## Appendix A Supplementary data

Multimedia component 1 **Figure S1**: Clinical signs of iridocorneal endothelial (ICE) syndrome. **(A)** Chandler syndrome. **(B)** Progressive iris atrophy. **(C)** Cogan-Reese syndrome. **(D)** Gonioscopy evaluation of peripheral anterior synechiae. **(E)** Corneal endothelium specular microscopy of normal fellow eye. **(F)** Corneal endothelium specular microscopy of ICE syndrome eye. **Figure S2**: TRAPD analysis of iridocorneal endothelial (ICE) syndrome. **(A)** Ancestry analysis of our ICE cohort. **(B)** Overall mutational burden of coding and noncoding segments. **(C)** Mutational ratio in the coding area of each patient. **(D)** No inflation was found with synonymous mutations (max gnomAD AF ≤ 0.01) in the ICE cohort versus the gnomAD cohort. **(E)** No inflation was found with rare benign noncoding mutations (max gnomAD AF ≤ 0.001; not predicted deleterious by any of the 3 algorithms: GERP++, GWAVA, and CADD). **Figure S3**: Epigenomic alterations of iridocorneal endothelial (ICE) syndrome. **(A)** Sample-level summary of hypomethylated region (HMR) frequency in 48 independent ICE samples. The bar plots show HMR counts within genomic features (HMR count), and counts of HMRs overlapping with CpG islands, shores, and shelves (CpG overlap). Multimedia component 1
