# Supplementary material for: The genomic and epigenomic landscape of iridocorneal endothelial syndrome
Source: Genes Dis. 2024 Nov 6;12(3):101448. doi: 10.1016/j.gendis.2024.101448 (PMC11919576; doi:10.1016/j.gendis.2024.101448)

Figure S1. Clinical features of ICE patients

A

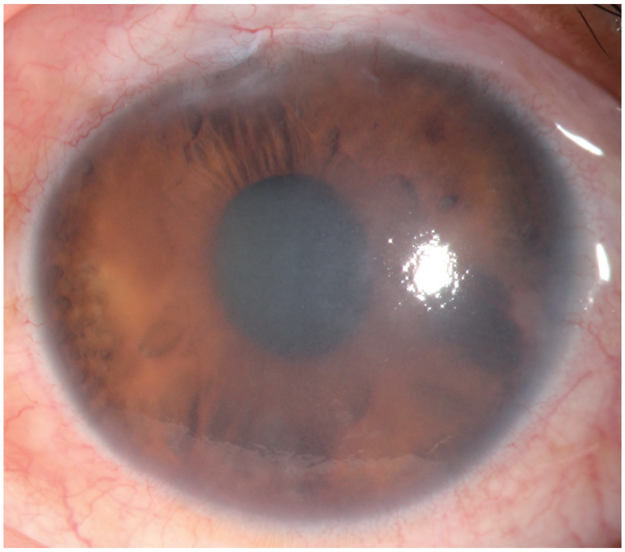

Chandler syndrome

B

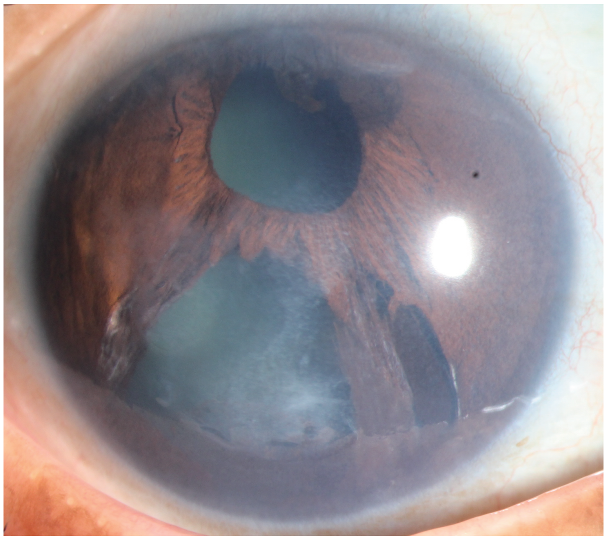

Progressive iris atrophy

C

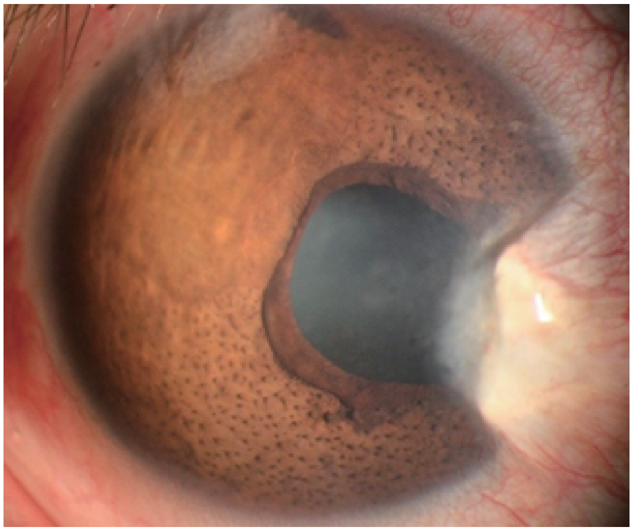

Cogan-Reese syndrome

D

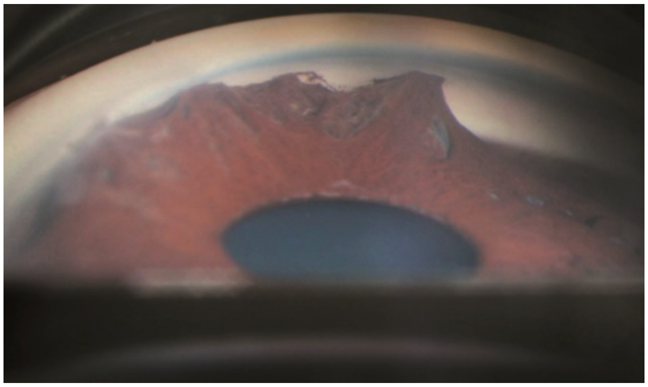

Gonioscopy evaluation of peripheral anterior synechiae

E

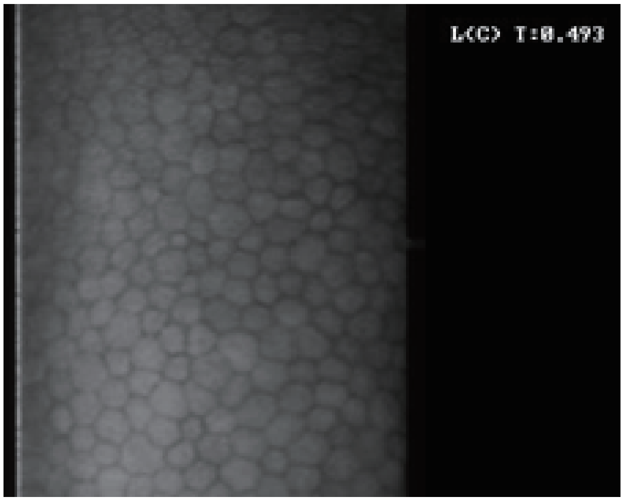

F

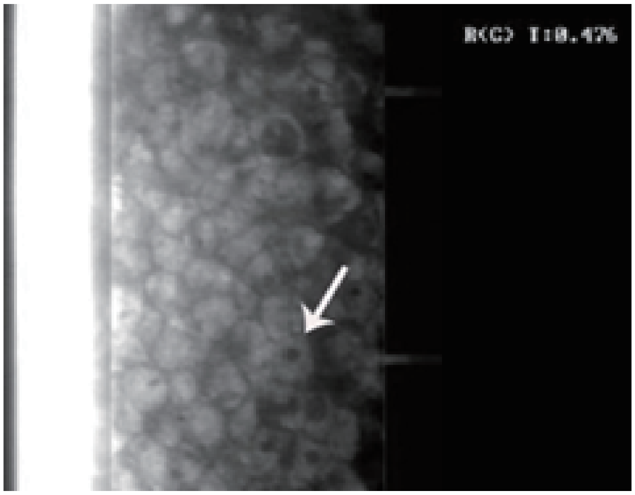

Figure S2. Region-based mutation burden analysis

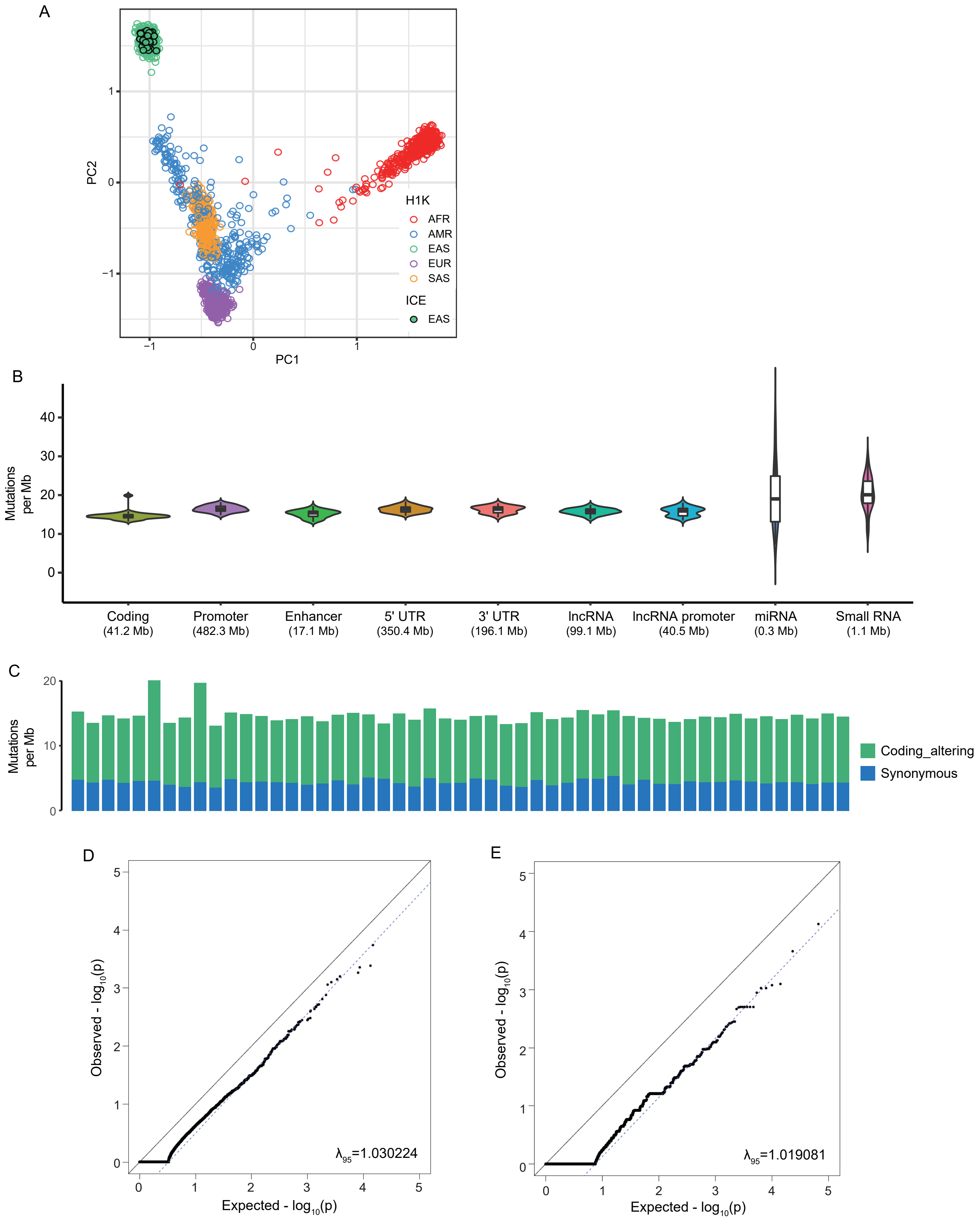

Figure S3. Epigenomic landscape of ICE syndrome

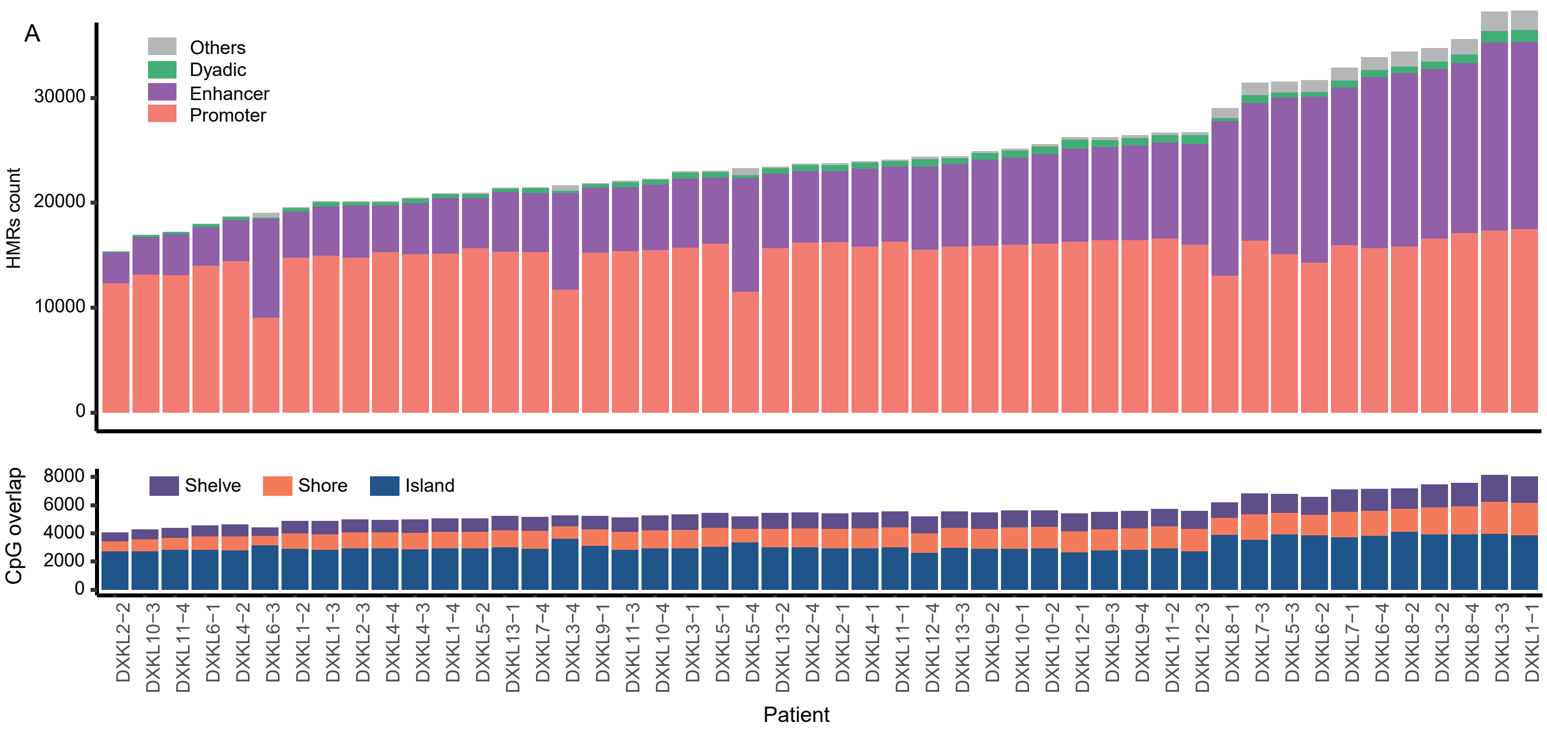

Supplement: Multimedia component 1 — Figure S1: Clinical signs of iridocorneal endothelial (ICE) syndrome. (A) Chandler syndrome. (B) Progressive iris atrophy. (C) Cogan-Reese syndrome. (D) Gonioscopy evaluation of peripheral anterior synechiae. (E) Corneal endothelium specular microscopy of normal fellow eye. (F) Corneal endothelium specular microscopy of ICE syndrome eye. Figure S2: TRAPD analysis of iridocorneal endothelial (ICE) syndrome. (A) Ancestry analysis of our ICE cohort. (B) Overall mutational burden of coding and noncoding segments. (C) Mutational ratio in the coding area of each patient. (D) No inflation was found with synonymous mutations (max gnomAD AF ≤ 0.01) in the ICE cohort versus the gnomAD cohort. (E) No inflation was found with rare benign noncoding mutations (max gnomAD AF ≤ 0.001; not predicted deleterious by any of the 3 algorithms: GERP++, GWAVA, and CADD). Figure S3: Epigenomic alterations of iridocorneal endothelial (ICE) syndrome. (A) Sample-level summary of hypomethylated region (HMR) frequency in 48 independent ICE samples. The bar plots show HMR counts within genomic features (HMR count), and counts of HMRs overlapping with CpG islands, shores, and shelves (CpG overlap). [file mmc1.pdf]
